# Upsetting experiences in the lives of neurodivergent young people: A qualitative analysis of accounts of adolescents diagnosed with attention‐deficit/hyperactivity disorder and/or autism

**DOI:** 10.1002/jcv2.70038

**Published:** 2025-08-19

**Authors:** Georgia Pavlopoulou, Susie Chandler, Steve Lukito, Myrofora Kakoulidou, Isabel Jackson, Elisa Ly, Maciej Matejko, Beta Balwani, Tiegan Boyens, Dorian Poulton, Luke Harvey‐Nguyen, Sylvan Baker, Edmund J. S. Sonuga‐Barke, the RE‐STAR team, Edmund Sonuga‐Barke, Susie Chandler, Andrea Danese, Johnny Downs, Eloise Funnell, Kirsty Griffiths, Myrofora Kakoulidou, Lauren Low, Steve Lukito, Umaya Prasad, Angus Roberts, Emily Simonoff, Daniel Stahl, Anna Wyatt, Georgia Pavlopoulou, Jane Hurry, Sylvan Baker, Graham Moore, Amy Edwards, Jessica Lennon, Dennis Ougrin, Amanda Roestorf, Rebecca Kirkbride, Claire Lewis, Jordan Altimimi, Beta Balwani, Saskia Barnes, Tiegan Boyens, Zoë Glen, C. J. Harris, Charlotte Hillman, Luke Harvey‐Nguyen, Isabel Jackson, Amber Johnson, Elisa Ly, Maciej Matejko, Dorian Poulton, Anya Rose, Darren Webb, Archie Wilson

**Affiliations:** ^1^ Group for Research in Relationships And NeuroDiversity (GRRAND) Research in Clinical, Educational & Health Psychology Division of Psychology & Language Sciences Faculty of Brain Sciences University College London London UK; ^2^ Anna Freud National Centre for Children and Families London UK; ^3^ School of Academic Psychiatry Institute of Psychiatry, Psychology & Neuroscience King's College London London UK; ^4^ University Hospitals of Leicester NHS Trust Leicester UK; ^5^ Cardiff University Cardiff UK; ^6^ Royal Central School of Speech & Drama London UK; ^7^ Department of Child & Adolescent Psychiatry Aarhus University Aarhus Denmark; ^8^ Department of Psychology University of Hong Kong Hong Kong China

**Keywords:** ADHD, adolescence, autism, emotional dysregulation

## Abstract

**Background:**

Accounts of emotional dysregulation in autism and attention‐deficit/hyperactivity disorder (ADHD) are typically based on external adult observations anchored in neurotypical notions of emotional responding. These often fail to place neurodivergent people's emotional responses in the context of the upsetting experiences they face; information best provided by the young people themselves.

**Methods:**

We interviewed 57 adolescents (11–15 years; 19 females) with diagnoses of ADHD (*n* = 24), autism (*n* = 21) or both (*n* = 12), about their experience of upsetting events using a co‐designed semi‐structured interview schedule. Reflexive thematic analysis generated shared themes with diagnosis‐specific nuances.

**Results:**

Four themes were extracted: social dislocation, alienation and conflict; need to mask; self‐doubt, loathing, embarrassment; and over stimulation/sensory mismatch. Upsetting experiences, for ADHD participants, were typically perceived as instigated by external agents trying to impose control, and/or a sense of injustice; for autistic participants they often related to feelings of ‘not belonging’ and alienation. Masking, for autistic participants, included ‘hiding’ negative emotions to protect others from their intensity; whereas in ADHD, masking usually involved supressing emotional upset to protect oneself from conflict or consequences. Those with a joint diagnosis reported a combination of these experiences, often felt more intensely.

**Conclusions:**

First‐person accounts of emotional responding could provide new insights with potential to refine current dysregulation‐based accounts of ADHD or autism.

## Key Points

- What's known: Existing accounts of emotional dysregulation in autism and attention‐deficit/hyperactivity disorder (ADHD) are typically based on external adult observations anchored in neurotypical notions of emotional responding. These often fail to contextualise neurodivergent people's emotional responses within the upsetting experiences they face.
- What's new: First‐person accounts of common upsetting events from 57 neurodivergent young people included four themes: social dislocation, alienation and conflict; need to mask; self‐doubt, loathing, embarrassment; and over stimulation/sensory mismatch. Autism‐ and ADHD‐specific nuances were found within these themes.
- What's relevant: First‐person accounts of emotional responding could provide new insights with potential to refine current dysregulation‐based accounts of ADHD or autism. This, in turn, could have implications for interventions which aim to promote the wellbeing of autistic young people and those with ADHD.

## INTRODUCTION

Adolescents diagnosed with attention‐deficit/hyperactivity disorder (ADHD) and/or autism, are at increased depression risk (Lai et al., 2019; Larson et al., 2011; Leyfer et al., 2006; Reale et al., 2017; Simonoff et al., 2008). Emotion dysregulation (ED), variously manifest as irritability, mood swings and outbursts, has been invoked to explain this (Cavanagh et al., 2017; Cisler et al., 2010; Cole et al., 2017; Fairchild et al., 2019; Joormann & Stanton, 2016). For instance, irritability has been proposed as a pre‐cursor of depression in ADHD (Eyre et al., 2017) and autism (Carter Leno et al., 2021). Traditional explanations have tended to take a deficit approach, focusing on neuro‐cognitive difficulties as mediators of the ADHD/autism‐to‐ED pathway. Bottom‐up difficulties in orienting to emotional stimuli (White et al., 2014) and reward processing (Bunford et al., 2015) and top‐down executive difficulties, such as reduced inhibitory control, have been linked to neurodivergent ED (Cibralic et al., 2019; Geurts et al., 2004; Groves et al., 2022; Shaw et al., 2014). Also implicated are emotion recognition difficulties, in relation to one's own and others' emotional states (Shushakova et al., 2018; Soler‐Gutiérrez et al., 2023) and limitations in metacognitive insights and skills, limiting emotion regulation strategies (Basile et al., 2021; Cai et al., 2020; Jahromi et al., 2012; Mazefsky et al., 2014).

*Regulating Emotion–Strengthening Adolescent Resilience* (RE‐STAR; https://www.kcl.ac.uk/research/re‐star) takes a different approach, shifting the focus from regulatory deficits within the individual to exploring the role of everyday emotional experiences in the pathway from autism and ADHD to depression—with the aim of developing more effective interventions to interrupt this pathway (Lukito et al., 2025; Sonuga‐Barke et al., in press). RE‐STAR is grounded in a participatory approach to translational science that places neurodivergent young people at its core, influencing all stages of the research process (Kakoulidou et al., 2024; Sonuga‐Barke et al., 2024). This in turn has led to a special focus on the role of context and experience in understanding ED in neurodivergence. Indeed, neurodivergent individuals have an increased exposure to emotionally upsetting events and encounters (Eccleston et al., 2019; Taylor & Gotham, 2016). They may also differ from neurotypical individuals in their reactions to such experiences (Eccleston et al., 2019; Mansfield & Soni, 2024). To date little research has examined which experiences and encounters are especially upsetting to neurodivergent young people. One study (Santomauro et al., 2017) involving seven autistic participants, identified events and experiences related to social interactions; school or employment; sensory overload; change; and fear of failure as especially upsetting. Garcia et al. (2019), found that arguments with parents about homework problems, personal hygiene, and bedtime are especially relevant in this regard. More recently, studies using participatory approaches have highlighted how camouflaging (e.g., the masking of autism/ADHD traits, or even mental health struggles), may be an additional stressor in the everyday lives of neurodivergent young people (Howe et al., 2023; McKinney et al., 2024; Rhodes et al., 2023).

Here we report on the experiences and situations that induce negative emotional reactions in neurodivergent adolescents aged 11–15 years old with a diagnosis of autism, ADHD or both. Our focus on the experience of neurodivergent young people is core to our strategy, acting as a corrective to prior studies that were reliant on measurement approaches constructed based on neurotypical understandings of emotion (Gratz & Roemer, 2004), and typically completed by clinicians or parent/carers (Mazefsky et al., 2018)—neurodivergent peoples' point of views being largely ignored (Cowen & Keltner, 2017).

## METHODS

### Ethics

**FIGURE 1:**
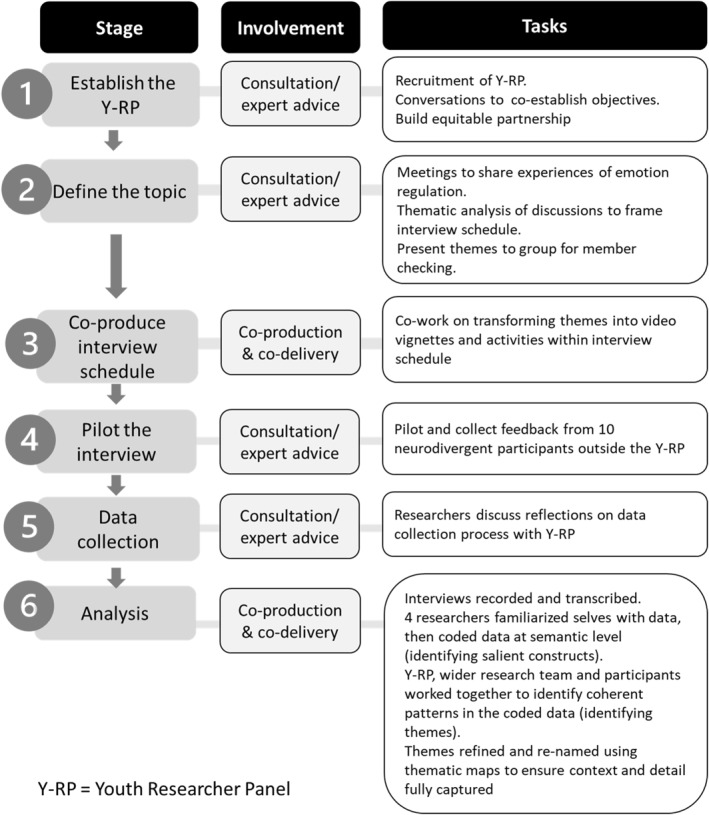
Stages of co‐production.

The study was approved by the Health and Social Care Research Ethics Committee A (HSC REC A; reference 22/NI/0017). Informed written parental/carer consent and young person assent were obtained for all participants. The current study was co‐designed with 10 young people between the ages of 18 and 25 years with diagnoses of ADHD and/or autism (Youth Researcher Panel (Y‐RAP); Sonuga‐Barke et al., 2024) who were paid for their time (NIHR, 2021). The collaboration between academic and youth collaborators was informed by an experience‐sensitive approach (McGreevy et al., 2024; Pavlopoulou, 2021) according to which youth co‐researchers are supported to contribute more fully to the research process, shaping its purpose and direction. Figure 1 illustrates the implementation of this approach.

### Participants

Fifty‐seven young people, aged 11–15 years, participated in the interviews. All attended UK mainstream secondary schools. Each had sufficient English to participate in the interviews. They were recruited via local National Health Service (NHS) clinics and national charities' networks via newsletters/social media. Eligibility criteria included: age (11–15 years 11 months at recruitment); diagnosis (a validated clinical diagnosis of autism and/or ADHD); attendance at a mainstream secondary school in England; sufficient use and understanding of spoken English to participant in the interview. Participants were assigned to the following groups based on their clinical diagnosis: ADHD (*n* = 24); Autism (*n* = 21); ADHD + Autism (*n* = 12), hereafter referred to as ‘dual diagnosis’. The sample was 67% male, and 75% white. 16% received free school meals (see Table 1 for sample characteristics).

**TABLE 1:** Sample characteristics.

	ADHD (*n* = 24)	Autism (*n* = 21)	ADHD + autism (*n* = 12)
Mean age in years (SD)	13.0 (1.40)	12.9 (1.35)	13.2 (1.4)
Sex, *N* (%)			
Male	18 (75.0)	11 (52.4)	9 (75.0)
Female	6 (25.0)	10 (47.6)	3 (25.0)
Receiving free school meals, *N* (%)	5 (20.8)	2 (9.5)	2 (16.7)
Ethnicity, *N* (%)			
Asian/Asian British	1 (4.2)	0 (0.0)	2 (16.7)
Black/Black British/Caribbean/African	1 (4.2)	1 (4.8)	2 (16.7)
Mixed/multiple ethnic groups	2 (8.3)	3 (14.3)	‐
White	19 (79.1)	16 (76.2)	8 (66.7)
Other	1 (4.2)	1 (4.8)	‐

Abbreviation: ADHD, attention‐deficit/hyperactivity disorder.

### Materials

A qualitative interview schedule was co‐developed with the Y‐RP. They contributed to the interview schedule, created video ‘vignettes’ to provide interviewees with an example of upsetting events (to prompt them to talk about their own experience), and piloted a ‘creative task’. The ‘creative task’ was designed to encourage participants to think in different ways about emotional experiences and to provide an opportunity to discuss issues beyond a traditional question and answer approach which can encourage reliance on repetition of existing discourses on certain topics. For this, they were asked to think of objects, places, situations and people (i) they find emotionally calming, reassuring or comfortable, (ii) they find emotionally difficult, triggering or upsetting. They were asked to create something using creative writing, photography, lego, clay modelling or drawing, See Supporting Information S1: I, II, and III for the ‘Creative task’ prompts (Figure S1), examples of completed creative tasks (Figures S2–S5), and interview prompts and visual aid (Figure S6) respectively. The interview schedule was piloted with 10 neurodivergent participants after which minor changes were made to the procedures. No changes were made to the interview schedule itself.

### Procedure

Parent/carers of potential participants completed a brief online screening questionnaire to confirm eligibility for the study. ‘Eligible’ parent/carers were sent an information letter, consent/assent forms, and a visual guide on the study process. Parent/carers were asked whether their child was aware of their autism/ADHD diagnosis, the degree to which they understood and identified with their diagnosis, and what terms they preferred to be used when speaking about autism/ADHD.

#### Orientation session

This lasted 15–30‐min depending on participant availability. Parent/carers and adolescents met with the researcher via Zoom. Participants were introduced to the goals of the study and the idea of using creative tasks to illustrate upsetting experiences and related emotions. Participants were given a warm‐up activity and described the interview schedule. They were then invited to share their ideas around the topic and choose a medium (drawing, photos, poetry) for the creative task. At the end of each meeting researchers ensured participants were clear on how to generate their own creative task. Participants were then given 2 weeks to complete the creative task, prior to the interview session.

#### Main interview session

This also took place on Zoom, and lasted between 1 and 2 h 15 min (mean duration: 1 h 30 min [SD 16 min]) Parent/carers were requested to remain on hand in case their child needed assistance. The interview began with participants presenting their creative work. An adapted version of the ShowEd protocol (Catalani & Minkler, 2010; Pavlopoulou et al., 2022) was used to discuss the meaning of this work. Participants then answered questions while taking part in interactive activities such as picking vignettes which probed participants' ideas and feelings about situations they found emotionally challenging. The interview was used to examine provocations/triggers of negative emotions. Interviews lasted between 40 and 90 min. They were audio‐ and video‐recorded, with appropriate consent/assent, and transcribed.

### Analysis

Interview transcripts were analysed line‐by‐line using inductive, reflexive thematic analysis, following Braun and Clarke (2006, 2019) and Campbell et al. (2021). The first 10 interviews were coded by 4 researchers, 2 of them neurodivergent, to standardise coding practices. The rest of the interviews were split across two interviewers who independently coded them. Two Y‐RPers also coded a sample of transcripts of participants matching their diagnostic group membership. Next, the initial findings were presented to team of academic researchers and Y‐RPers. These gave feedback on emerging codes and their clustering. Codes were clustered into initial themes during group discussions between the academic researchers and Y‐RPers. The results were not structured according to the research questions, but rather to themes that appeared across the interviews as a whole. While all themes were relevant to neurodivergent young people regardless of their diagnosis, at times the interpretation and content of subthemes differed between the three groups. The team noted contrasts within each theme. A final set of themes was agreed through an iterative analytic process of multiple sessions between the academic researchers and Y‐RPers. Codes were discussed with a subsample of study participants (*n* = 6), who provided feedback on the near‐final themes, written in lay language. Final themes were discussed and refined, in both one‐to‐one and group meetings, by an interdisciplinary and neurodivergent team of psychiatrists and psychologists, both clinical and non‐clinical.

### Positionality and reflexivity

The subjectivity of the academic researchers and the Y‐RP was explicitly discussed during weekly and monthly meetings that were set up to discuss the self we bring in the research, recognising our familiarity with the research topic on both a scientific and personal level. This included both the different types of expertise and experience individuals brought to the research, as well researchers' and Y‐RP experience of neurodivergence. According to Morse et al. (2002) such an approach is superior to the use of ‘unknowledgeable’ coders, who may be less able to add richness that ‘insider’ researchers often bring to the coding process.

## RESULTS

The found themes, with example quotes, are presented in Table 2 and summarised below.

**TABLE 2:** Summary of themes with participant numbers and example quotes.

Theme	Example quotes
1: Social dislocation, alienation and conflict (57 participants)	‘… I had two friends. Two main friends, and I wouldn't say I was friends with them. I'd say there were more kind of bullies pretending to be my friend. And that left an absolute mark on how I treat friendships, just a complete dump on my mental health. So say, … we'd play a game kind of like handball … And since when I came to the realisation that these people, weren't, you know, really, my friends they were just like, it kind of initiated an unconscious thing that I've only really noticed now … Like he said as a joke “Nobody likes you” and that, even though I know it's a joke, absolutely hits me … My friendship trauma is long…’. (ADHD male, 14)
	‘I often been really excited about something and they (meaning friends) don't match my energy about it … it makes me feel a bit deflated and sad, because I feel a bit rejected. And that will really affect me. I'll just be really sad and fed up … That's a big one. (Female, 15, Autistic) If I got quite angry, it's probably because someone was annoying me and like they weren't listening. They would just be like – they would just not be listening if I was telling them something, like, important or something, and they weren't listening, and I had asked them to listen and it was important’. (Autistic female, 12)
	‘There are some of my friends who … they're like “You're not disabled, though. You aren't in a wheelchair. You haven't got a broken leg, nothing like that”. But I'm like “I'm disabled still” and they're like “But you're not.” And that makes me a bit upset … because they don't believe me, they're actually saying I'm a liar for saying I'm disabled when they're saying I'm not. But why would I lie about being disabled? That's the thing that's confusing and makes me upset because they're not understanding me properly …’. (Dual diagnosis male, 14)
2: Experience of hiding one's true self (46 participants)	‘I don't want to show that always. At school, mainly, I don't show it as much, because I can probably get in a lot more trouble at school. So at school, I'll be very angry inside. I just won't show it. Where at home, I can probably show my anger a bit more because there are less restrictions of maybe getting a detention or something that you can't get at home’. (ADHD male, 12)
	‘Hide it, … I just prefer it to stay between me instead of me causing a problem’. (Autistic male, 13)
	‘Well, it's all bubbled up inside me, it's getting stronger, stronger, stronger, a bit like a cloud. All the water's inside and then it just bursts. I think that's the same as anger in me’. (Autistic male, 12)
	‘… if I just show myself who I actually am to begin with, I might put like a bad impression or think I'm some just some kid with like an issue or something. Because … I don't want them to think straight off the bat, I just want them I'm just a chill person … I'll just keep everything to myself’. (Dual diagnosis male, 15)
3: Self‐doubt, loathing, embarrassment (51 participants)	‘People won't understand and then it makes me like, annoyed and a bit angry because I'm not the clearest person … It can be quite frustrating’. (ADHD male, 13)
	‘I'm not really that clever cause I don't listen in school because it's hard for me to listen when I'm always moving or when I'm always not listening or going in my own world … I'm like, not as clever as the others’. (Autistic male, 11)
	‘I kind of feel rejected and a lot of negative thoughts go through my head, like I don't really deserve much. Like such as I don't really deserve affection or love’. (Autistic female, 15)
	‘It's kind of gotten normal because I'm quite annoying. So whenever they ignore me, it's like, okay, time to shut up now … So it's mainly all my fault, really … That's how to describe me in one word. Silly … I make stupid things’. (Dual diagnosis male, 11)
4: Over‐stimulation/sensory mismatch (47 participants)	‘Because, mainly at the times where I don't get it, that makes it a bit more boring, because I don't get what's going on. And then it makes me a bit tired …’. (ADHD female, 13)
	‘When I'm doing like, things that are just not really exciting. I just make me feel make me feel like so – quite down they're not, like, very – I don't really know how to explain it. It's just boring’. (ADHD female, 13)
	‘I get stressed over things being squished into one small area, and claustrophobic actually. I fear small spaces because it makes me, it just makes it hard to breathe. And I've been like this for a long, long time’. (Autistic male, 12)
	‘It's just people interrupting my lessons irritates *me* and agitates me’. (Autistic male, 13)
	‘If someone interrupts me, then it makes me frustrated. Not really, it's normally when I'm just doing something I like and then I get called to do something. I normally either don't respond, and when I do get called to do something, then I get quite frustrated. Because, when my focus is broken from something that I like, it sort of breaks the immersion’. (Dual diagnosis male, 12)

In the following section, all themes will be discussed in turn, highlighting variation between participants within subthemes, where appropriate.

### Theme 1: Social dislocation, alienation and conflict

All participants described a marked sense of social unease during, as well as when they were excluded from, everyday interactions. This led to negative feelings about themselves and others, particularly when this was initiated by neurotypical people.

… I didn't know why these people didn't like me … I feel like I don't have a concrete friend group, at all. It really does hurt sometimes … That I'm gonna die alone, or without any friends, or I'm gonna die single. Those fears haunted me pretty much every single day … I feel like the extra friend a lot … It feels horrible … It gets under my skin every single time.

(Female, 15, autism)

All noted times they were dismissed or ignored by others and that this can create frustration and negative thoughts:

I hate being ignored or I hate being dismissed by friends … When I get ignored, it makes me feel like I'm really hot and sweaty and like it just works me up.

(Female, 15, ADHD)

(even if ) they sometimes understand … they do not normally relate to me.

(Female, 13, dual diagnosis)

Feelings of alienation and/or being seen by others as a problem were not always discernible as discrete provocations in the young people's quotes—but rather more as an ongoing experience that related to feeling angry, damaging property or crying.

#### Stigma, conflict and victimisation

A central subtheme in the narratives of autistic participants with or without ADHD diagnosis was the paradoxical feeling that they attracted too much attention because of their diagnostic label, whilst feeling invisible, or even being perceived as rude, when expressing their views and needs. Participants emphasised how the stigma attached to autism undermined their confidence and self‐esteem and left them feeling sad and lonely:

When people sum autism up as a negative thing, it makes me feel like I am worthless. When people say it’s something that needs to vanish, I feel like I am an odd species, speaking an unknown language. How am I supposed to feel confident with these thoughts around me? How am I supposed to happily unfold? It slowly starts to hurt me. You can’t fix me ‘cause there is nothing wrong with me and … the only thing to fix is the world’s perspective’.

(Female, 12, autism)

Notably, those with a dual diagnosis referred to incidents of mismatch and conflict with authorities within the school environment provoking feelings of rage:

Another thing that makes me get quite angry is the teacher shouting. It happened today, in the last lesson, because the class was being really loud, and he shouted out “Be quiet”. And then that sort of just tipped me over the edge.

(Male, 12, dual diagnosis)

Recollections related to victimisation and unsafe, upsetting interpersonal interactions were common, most frequently in the accounts of those with an ADHD diagnosis.

I probably feel like a bit panicky or a bit like everyone's looking at me. Am I going to get beaten up? Because girls are just really like, girls just can be like proper, like, horrible people sometimes. But I kind of just walk away to get away from the situation or prove I didn't do it really.

(Female, 15, ADHD)

Descriptions of incidents of conflict with authorities within the school environment provoking feelings of rage were common for all participants.

Those with an ADHD diagnosis viewed their anger as an attempt to get ‘justice’ for themselves or others or indignation when misled or made to look foolish:

If I am asked to do something, I want to know why … sometimes people don't want to explain why they want me to do things … Unfairness upsets me.

(Male, 14, ADHD)

I just needed to find out why she [the teacher] called me rude, because I hadn't been rude and why it [fidget toy] needed to be taken off me because I wasn't distracting anyone.

(Male, 13, ADHD)

#### A mismatch of salience

Most participants with an ADHD diagnosis described how others found it difficult to interpret or tolerate their levels of excitement (e.g., when anticipating a positive event). For example, ‘*It might be a bit stressing for them to deal with*’ (Male, 11, ADHD) and the young person may ‘*get in trouble*’ (Male, 13, ADHD) because they ‘*can't stop being excited*’ (Male, 15, ADHD).

This could provoke feelings as diverse as guilt or rage.

I told them it was important, and they just didn't listen, and I would get kind of angry at them and mad at them.

(Female, 12, autism)

Some participants also noted that is often hard for neurotypical adults and peers to empathise and that sometimes feels like others may be ‘*acting like they don't know why they made me angry*’ (Male, 11, dual diagnosis) and that this mismatch can create frustration and anger.

I feel like I'm a caged monkey, and just something to look at because of how unusual it can act. And I don't like that, because I'm like a caged monkey when they don’t understand me.

(Male, 13, autism)

### Theme 2: The experience of hiding one's true self, the need to mask

Participants told how they regularly hid their ‘natural’ feelings, preferences, and reactions. This, in turn, provoked feelings such as sadness and ‘burnout’, which sometimes led to ‘meltdowns’. Participants gave different reasons for masking. Some suggested that expressions of their true emotions might be misunderstood as attention‐seeking, mischaracterised as ‘dramatic or annoying’ (Female, 12, autism) or them being a considered a ‘cry‐baby’ (Female, 15, autism).

I don't want it to feel like that I'm kind of trying to get people's attention or using it as an excuse or using it kind of to make people feel sorry for …. I don't like telling people it because I don't need attention.

(Female, 12, autism)

#### Efforts to protect others from my feelings

Autistic participants described masking as an effort to protect others from the intensity of their emotions and to minimise the emotional burden for others which triggered shame and neglecting self‐care.

If I express stress …., people around me might get stressed … So I think it's just better if I don't tell people and then they don't have to worry about me … I don't want to make it an issue for someone else ….

(Male, 13, autism)

She [her sister] constantly worried about me that there was something wrong with me. … I had to hide all my feelings from her … Just feels like sad because I want to be able to express …. I don't want to, like, be a burden or anything.

(Female, 13, autism)

Participants with dual diagnoses reported feelings of guilt and confusion triggered by hiding their true self.

So how I feel is that when I can't fully express my emotions to people outside of my family because they will judge me, they'll make me feel as if I'm in the wrong for feeling a certain way.

(Male, 12, dual diagnosis)

I do think I definitely mask a lot at school. I mask a lot of my feelings, of how I feel. Especially to teachers as well, if they ask me if I'm okay, I'll say, Yeah, I'm fine.

(Female, 15, dual diagnosis)

Which then turn into anger.

I bottle it up, if that makes sense (…) I just – I push it inside. And I just bottle it up, put a fake mask on. (…) it's like I explode, ‘cause I bottle up so many emotions (…)’ It will be like a volcano; I'll bottle loads of things up, and when I get angry, I'll just [imitating explosion].

(Female, 13, dual diagnosis)

#### A constant need to protect self from others' reactions

Some participants with a diagnosis of ADHD reported masking as a ‘choice’ to protect themselves from ‘arguments and turmoil’ (Male, 13). In their experience, it was not safe to express emotion and by keeping ‘emotions locked away’ (Female, 13) they were less likely to look like ‘a fool’ (Female, 13) to peers.

I don't feel good masking them, but it's better than actually showing my feelings and getting made fun of, me getting angry and maybe get a detention or …. to be seen as a wimp.

(Male, 13, ADHD)

This often led to feelings of rage and was eventually escalated to behaviours of concern (kicking, hitting etc).

It can start off like lighting a match and then it burns. It burns and it just goes bang and I let it all out in ways like, and I know I shouldn't and I'm not happy that I do but shout and I punch the wall and stuff.

(Male, 13 years, ADHD)

### Theme 3: Internalised self‐doubt, ‐loathing and embarrassment

Participants described how they struggled to keep up with the neurotypical standards they felt were expected of them. They described emotions of anger, sadness and or apathy/giving up triggered of these negative feelings.

I'll feel like I've disappointed myself, I'll feel like I've done bad for myself, like I've caused a problem or I've disappointed other people than myself.

(Male, 13, autism)

These feelings were linked, in turn, to low self‐esteem; impacting not just the way the young people thought about themselves in the present, but also their future:

Just thinking about what could happen if I don't understand or do stuff properly, the repercussions that will have in the future, which then stresses me out a bit more.

(Female, 15, autism)

Participants also described getting into trouble in the classroom because of their different ways of attending. For instance, because it was different from neurotypical attention. They also tended to take responsibility for things going wrong, and often ‘apologise a lot of times’ (Male, 13, autism).

I'm just scared that I'm going to say the wrong thing. In the sense of that, I'll pick up the wrong piece of information (…) I'm scared that they are going to be like ‘You lied to me’, or something like that.

(Male, 15, ADHD)

Repeated past experiences of negative/confusing social interactions were found as key triggers to explosive behaviours as participants admitted they may ‘get emotional really quickly with that one thing’ (Male, 15, ADHD) since the negative memory ‘stays in the back of my mind so I can be angry at somebody when I see them, the anger just comes out’ (Female, 13, ADHD). The fear of re‐living a painful experience, even in a completely new and safe situation with a completely new person, was then an additional trigger, due to the times they ‘have been ignored before’ or someone else ‘made fun’ of them (Female, 13, autism).

#### Invisible and dismissed

For autistic participants, worries were triggered by self‐criticism and self‐doubt relating to social situations.

With my friends, I just worry that they're not going to forgive me or something. Even if it's like the smallest thing, I stress about it.

(Female, 12, autism)

It's more after I feel the regret, Why did I do that? Why did I like? Why did I – like I can do some really stupid stuff. I always regret it after….

(Female, 15, autism)

I just felt like they didn't care about how I felt or my significance or anything or my voice.

(Female, 13, autism)

They would just be like – they would just not be listening if I was telling them something, like, important or something.

(Female, 12, autism).

These participants described themselves as feeling invisible to others around them, making it harder to justify and protect themselves when others dismiss their emotional experience by saying for example, that there is ‘nothing really going on to make me sad’ (Male, 12, autism). This, in turn, multiplies the young people's self‐doubts and internal shame, often expressed with low mood or neglecting self‐care.

#### Guilt and embarrassment when help is needed

Those with a dual diagnosis reported feeling particularly guilty and embarrassed because of their need to ask clarification or help.

I feel guilty because I keep asking questions.

(Male, 14, dual diagnosis)

I feel quite embarrassed I just walked into someone or just about to.

(Male, 15, dual diagnosis)

Like a bit embarrassed because I didn't realise that I was doing it badly.

(Female, 12, dual diagnosis)

Also, they mention that the moments they need help may be particularly triggering due to memories from times they felt embarrassed or annoyed or help was denied.

Furthermore, participants with a diagnosis of ADHD expressed that they felt did not deserve accommodations as ‘*it's not really fair on other people or me to be treated differently*’. (Male, 13, ADHD). Such negative internalised beliefs seemed to create ‘internal stress’ (Male, 11, ADHD) that reinforced negative thoughts and self‐critical thinking patterns that then allowed ‘sadness to kick in’ (Male, 13, ADHD).

… It annoys me because I know everyone else with normal – without mental disabilities, it's easy for them … It will be in their head like […] it's hard for me to get all this knowledge out and use the knowledge.

(Male, 13, ADHD)

### Theme 4: Under/over stimulation and sensory mismatch

Participants agreed that sensory input and stimulation levels often trigger negative emotional responses ranging from frustration and anger to feeling scared, worried and sad. Notably, most participants felt more in control of sensory input at home and most quotes around provocations reflected situations occurring at school. Most autistic participants who also had an ADHD diagnosis described experiences of sensory overload as trigger for confusion, rage or panic.

Being in a noisy place is one of the worst that I seem to experience at the moment. My ears are extremely sensitive. So even just slight noise is bad.

(Female, 12, dual diagnosis)

#### Over‐stimulated

Autistic participants talked about their internal need to reduce stimulation which can trigger emotion responses that may look like irrigatable or controlling behaviour.

(…) if I'm trying to focus on something and then there's kind of noises in the background, they will really irritate me. Or if I'm really anxious by something, and then there's that extra noise and things like, I can be very irritable with, like, all my senses really.

(Female, 12, autism)

Auditory overload was associated with provoking ‘meltdowns’ and ‘burnouts’ associated with feelings of being scared:

[in the school corridor] it is very loud and crowded and there are people twice my height barging past me like I’m an insignificant insect. People shout at each other and push each other into the banisters on the stairs and I have almost fell down the stairs multiple times and it is very scary.

(Female, 13, autism)

Autistic participants also described how unexpected sensory stimulation could interrupt their ‘flow’ during an activity just at the time they may have become ‘really interested’ (Female, 15). These intrusions were reported not only as triggers for irritability and nervousness but also to exaggerate existing emotional struggles.

#### Under‐stimulated

The ADHD group reported that anger and boredom were more likely to be experienced when they are feeling bored or under‐stimulated.

During school [I experience big emotions], mostly, because what we do now is just going over the stuff we've already learned which is just a bit boring, the repetition. And then I guess when a lesson or doing something is boring and I just switch off. And that can make me tired … my mind will just wonder. And like, I'll wake up ten minutes later, and Miss is like, Okay, do the questions now, and I've just been sitting in my head, just thinking about games, films, shows.

(Male, 15, ADHD)

## DISCUSSION

This study provides the first large scale, in‐depth qualitative exploration of everyday upsetting events from the perspective of neurodivergent young people. Our findings highlight the importance of taking the young person's point of view into account when trying to explain neurodivergent emotion. Four findings were especially noteworthy.

First, our results give nuance and depth to some concepts central to prior accounts of neurodivergent emotion (Conner et al., 2020; Seymour et al., 2014). For instance, all participants described frequent and intense relational provocations as part of their everyday lives. However, while the autistic group tended to focus on social exclusion and stigma, the ADHD group focused more on the conflictual nature of interactions and a deep sense of injustice, as a key provocation. The dual diagnosis group made more references on triggers related to school interactions and demands. Masking or suppression of emotional responses was a common theme in all three groups' accounts, supporting previous findings (Howe et al., 2023; McKinney et al., 2024; Rhodes et al., 2023). However, where previous studies have focused on ‘fitting in’ or assimilation (McKinney et al., 2024) and stigma avoidance (van der Putten et al., 2024) as the key motivation for masking, our results go further to suggest that motivations for masking can be varied for autistic versus ADHD young people. Further, the autism group, similarly to dual diagnosis group, described the ongoing pressure to mask as a burden and a trigger for negative emotions, while the ADHD group framed masking as a ‘response’ to other types of provocation in order to avoid being made fun of or getting into trouble. All three groups highlighted the impact that struggling to adhere to neurotypical norms of behaviour had on their self‐worth, emotional regulation and wider mental health. Sensory and stimulation triggers were reported to be overwhelming and distressing for all participants. However, while the ADHD group described unexpected sensory stimulation or low levels of stimulation as an emotional trigger, the autistic young people and those with dual diagnosis underlined particularly sensory overload as a trigger.

Second, our analysis challenges some core elements of existing accounts. For instance, reflecting on traditional models of divergent emotional reactions in autism and ADHD that ascribe these to internal difficulties in self‐regulation (e.g.,Mazefsky & White, 2014; Shaw et al., 2014)—this view was not validated by, nor resonated with the experiences of the young people in the current study. For example, the young people rarely mentioned concepts such as dysregulation, or feeling out of control. When they reflected on the externalised manifestations of their emotions such as outbursts and shutting down, they framed them in different ways. For instance, for some there was a sense that their emotional responses were not the product of a failure to regulate but rather were a way of expressing, handling or masking an emotion caused by repeated environmental triggers—they had a functional element.

Third, some themes may be in stark contrast to claims made in previous ADHD and autism research. For instance, dominant autism theories (e.g., theory of mind, mind‐blindness, empathising‐systemising (Baron‐Cohen, 1997; Baron‐Cohen et al., 1985)), propose that autistic young people have limited interest in developing relationships with others as well as an ‘impaired’ reflective ability to think about other people's emotions and the consequences of their actions. However, a failure to listen to the autistic person's own accounts may have led to potential mischaracterisation of autistic people as lacking empathy (Fletcher‐Watson & Bird, 2020). Indeed, our findings provide indirect evidence for the presence of empathy among the autistic participants, who described masking as a learnt strategy to avoid upsetting or causing concern or emotional distress in others.

Finally, negative emotional experiences of youth common in the general population (Compas et al., 1987) are identified here as specifically relevant to neurodivergent youth. Descriptions of triggers were dominated by inter‐personal provocations, feelings of not belonging and social injustice. Self‐doubt was also very common. Mental health is increasingly recognised as a systemic issue in the general population, with a wide range of contributing and interacting factors (Cohen, 2017). However, it is possible that the focus on internal deficits as the source of neurodivergent emotional responses has meant that little attention has been given to the social determinants that manifest in the emotional lives of neurodivergent individuals (e.g., through feeling ‘othered’ or discriminated because of neurodivergence).

The accounts reflected elements of a general stress reactivity model of mental health (Ingram & Luxton, 2005), describing how ongoing and intense stress in the environment (e.g., sensory or social triggers) can reduce one's capacity to manage challenging situations. Many of the identified themes that refer to relational and environmental stressors have been identified as triggers for low mood (Kehusmaa et al., 2022). However, these have rarely been a focus in the ADHD and autism literature. This study is consistent with previous work (Gibbs et al., 2023) reporting that neurodivergent young people experience emotional provocations more frequently and find them very upsetting due to their chronic and repeated nature. Our data suggests negative environmental experiences (e.g., social and sensory) impact not only on how neurodivergent young people think about themselves but also how they approach new challenges.

### Strengths and limitations

The study has several strengths. First, it had a large sample for a qualitative study. Second, it included three diagnostic groups, often studied separately. Third, the study was co‐produced with the Y‐RP who offered valuable input as active co‐researchers across all research phases. Fourth, it employed novel interactive, arts‐based approaches to eliciting information about emotion from neurodivergent adolescents. The main limitation was the lack of representation among non‐speaking neurodivergent young people, those with intellectual disabilities, those from global majority or those attending special school/not attending school; we do not know the extent to which our findings apply to these groups. Additionally, we do not know how many participants had additional co‐occurring neurodevelopmental, physical or mental health conditions, and how these may have influenced their everyday experiences, exposure to triggers and emotional responses.

## CONCLUSION

Our study gives important insights into the emotional lives of young people with diagnoses of ADHD, autism or both. In doing this it highlights how important it is to include considerations of the specific nature of environmental provocations when designing mental health interventions. A holistic approach Is called for where, on the one hand agencies work together to adapt environments to reduce provocations where they can (e.g., in the classroom) and on the other they work with young people to manage the challenges faced in their day‐to‐day lives. The goal being to foster positive development by supporting self‐worth and growing agency and belonging (Pavlopoulou, 2021; Sonuga‐Barke, 2023).

## AUTHOR CONTRIBUTIONS

**Georgia Pavlopoulou**: Conceptualization; formal analysis; funding acquisition; methodology; writing—original draft; writing—review and editing. **Susie Chandler**: Formal analysis; project administration; resources; writing—original draft; writing—review and editing. **Steve Lukito**: Formal analysis; writing—review and editing. **Myrofora Kakoulidou**: Data curation; formal analysis; methodology; writing—original draft; writing—review and editing. **Isabel Jackson**: Formal analysis; writing—review and editing. **Elisa Ly**: Formal analysis; writing—review and editing. **Maciej Matejko**: Formal analysis; writing—review and editing. **Beta Balwani**: Formal analysis; writing—review and editing. **Tiegan Boyens**: Formal analysis; writing—review and editing. **Dorian Poulton**: Formal analysis. **Luke Harvey‐Nguyen**: Formal analysis. **Sylvan Baker**: Writing—review and editing. **Edmund J. S. Sonuga‐Barke**: Conceptualization; funding acquisition; methodology; resources; supervision; writing—original draft; writing—review and editing.

## CONFLICT OF INTEREST STATEMENT

The authors declare no conflicts of interest.

## ETHICAL CONSIDERATIONS

Informed consent/assent was appropriately obtained (or parental consent if required) prior to participation. The study was approved by the Health and Social Care Research Ethics Committee A (HSC REC A; reference 22/NI/0017) on 8 February 2022. Informed written parental/carer consent and young person assent were obtained for all participants.

## Supporting information

Supporting Information S1

## Data Availability

The data that support the findings of this study are available from the corresponding author upon reasonable request.
