# Supplementary material for: Upsetting experiences in the lives of neurodivergent young people: A qualitative analysis of accounts of adolescents diagnosed with attention‐deficit/hyperactivity disorder and/or autism
Source: JCPP Adv. 2025 Aug 19;6(2):e70038. doi: 10.1002/jcv2.70038 (PMC13260670; doi:10.1002/jcv2.70038)
Supplement: Supplementary file 1 — Supporting Information S1 [file JCV2-6-e70038-s001.pdf]

SUPPORTING INFORMATION

I. CREATIVE TASK PROMPTS

Participants received the following information on how to create something to describe their emotions (excerpt from participant information sheet below):

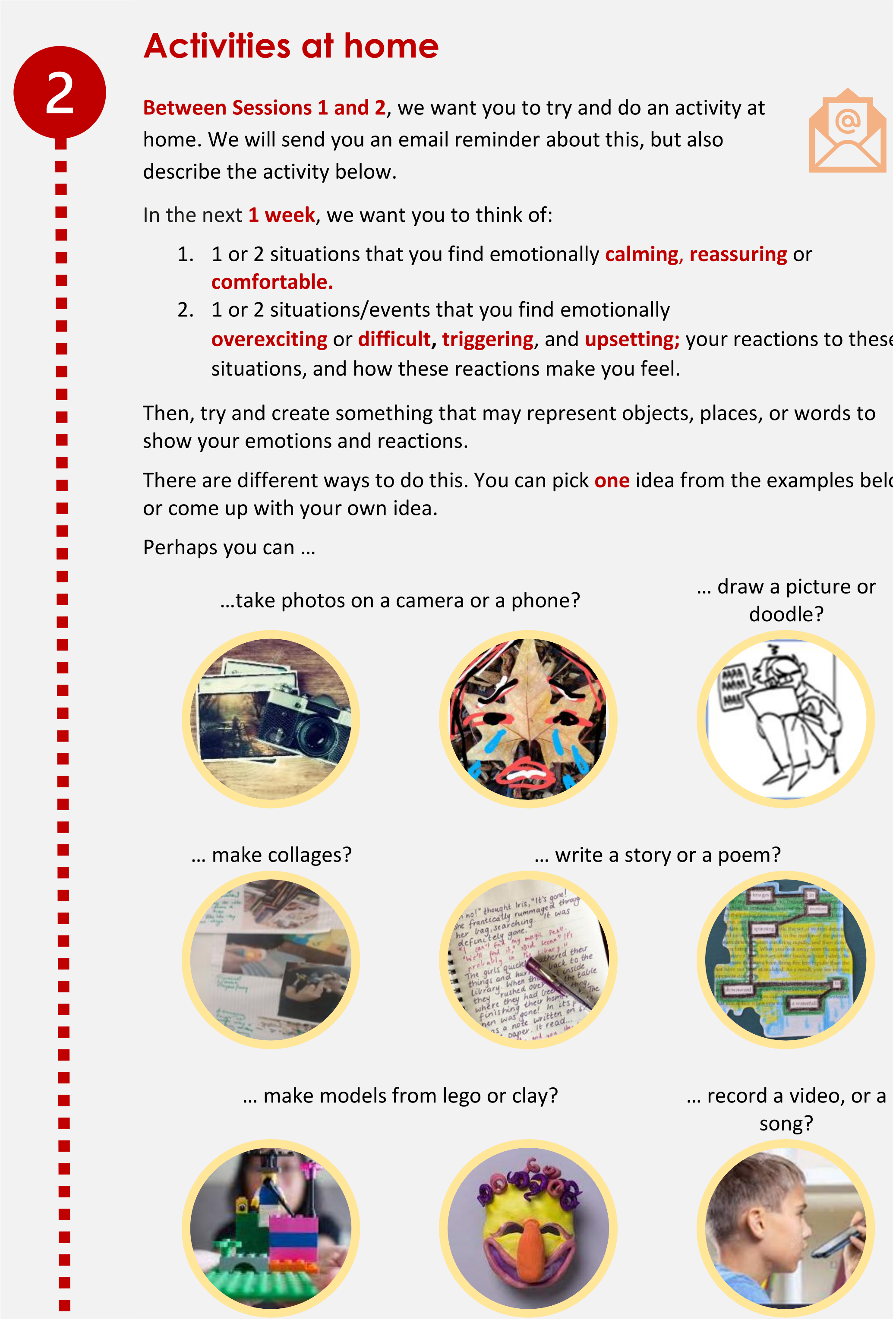

Figure S1. Instructions provided to participants for the creative task

Correspondence to: georgia.pavlopoulou@ucl.ac.uk ; edmund.sonuga-barke@kcl.ac.uk

II. EXAMPLES OF CREATIVE TASKS COMPLETED BY YOUNG PEOPLE

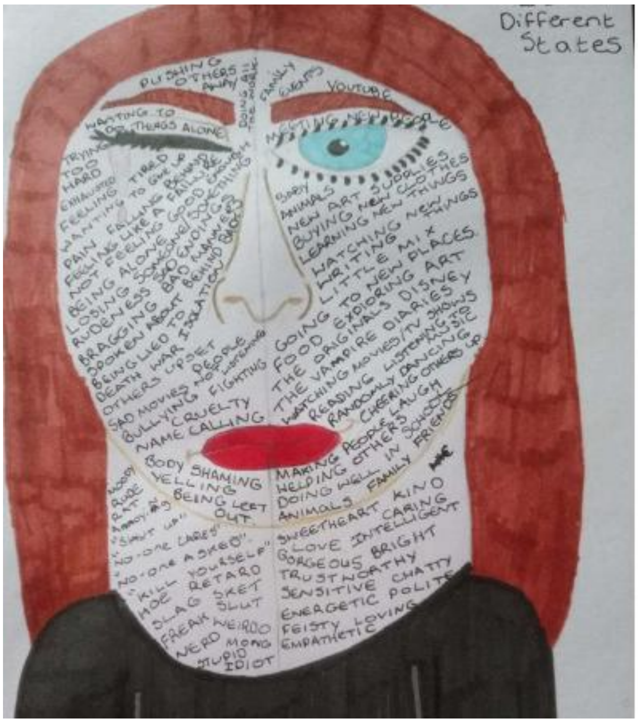

Figure S2. Drawing describing “different states” (Age 14, female, ADHD).

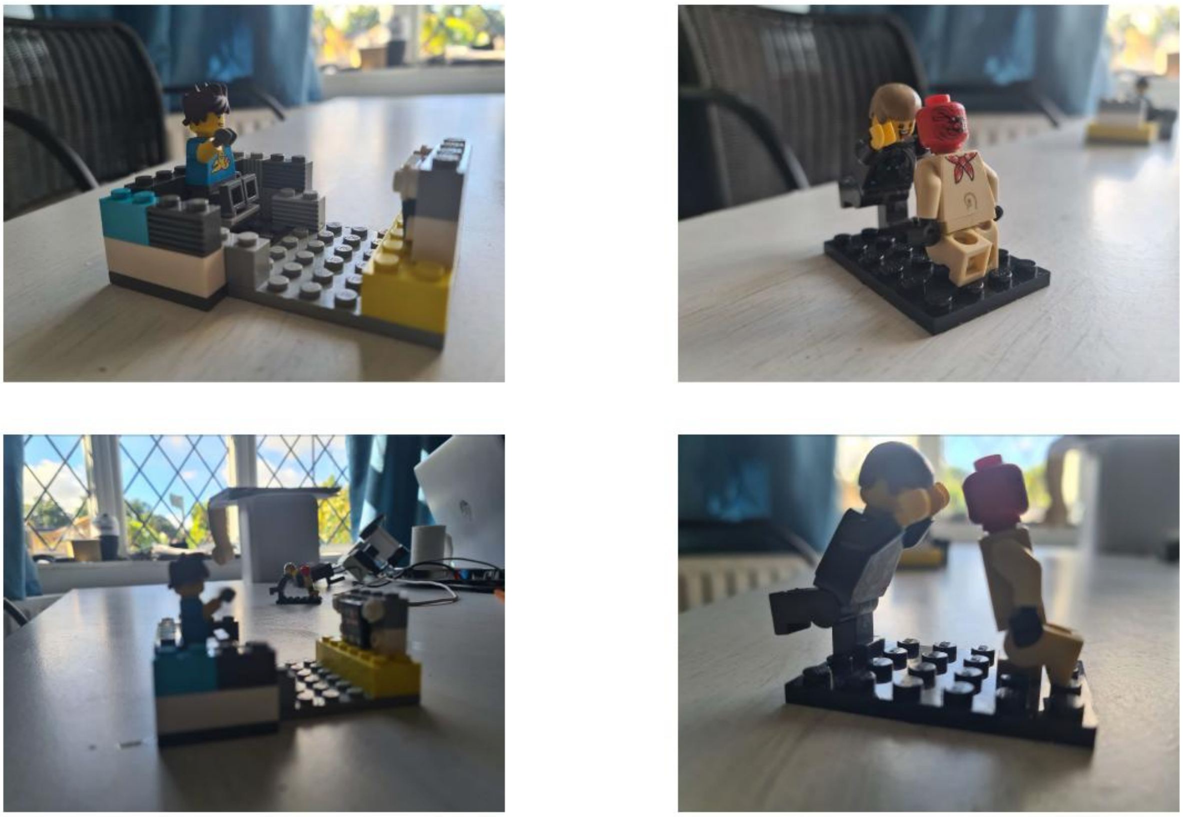

Figure S3. Photos of Lego models representing different emotions (Age 13, male, ADHD)

Correspondence to: georgia.pavlopoulou@ucl.ac.uk ; edmund.sonuga-barke@kcl.ac.uk

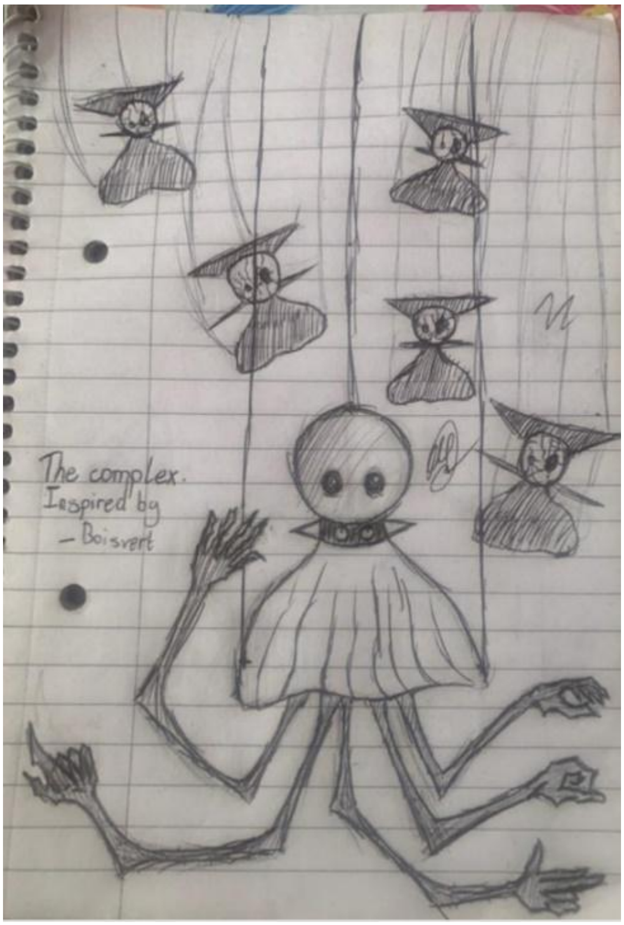

Figure S4. “You can see the people behind me there to help me. They're all packed in, and they're all kind of the same size or distance, showing how I get stressed over things being squished into one small area, and claustrophobic actually” (Age 12, male, autism).

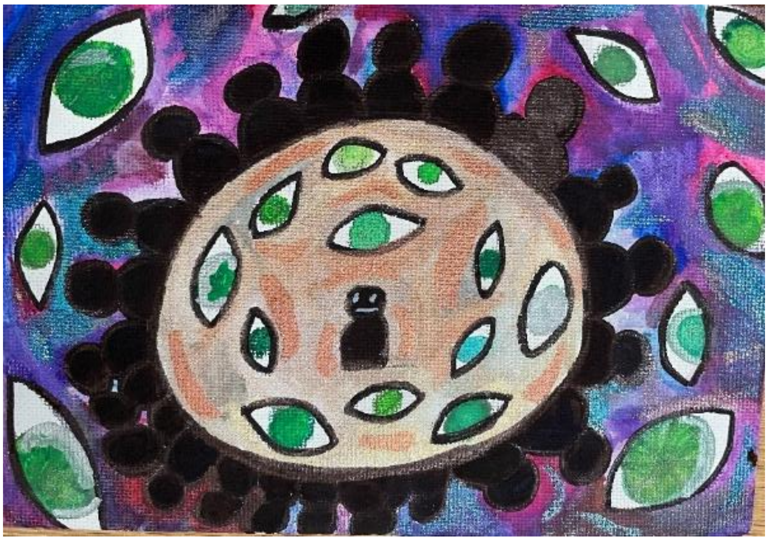

Figure S5. “You just feel like somebody is glaring at you. It might not be like in a bad way. And then if you … do something wrong, then everybody's going to turn on you.” (Age 14, female, autism).

Correspondence to: georgia.pavlopoulou@ucl.ac.uk ; edmund.sonuga-barke@kcl.ac.uk

III. INTERVIEW SCHEDULE

Thank you so much for taking part in this interview. Today, we will go through different topics, and look at anything you have created for today's session. In particular, we would like to have a chat about your experience of strong emotions and how you manage them in different situations.

We will not share what we talk about today with your parents or anyone else unless we are concerned about your safety.

If there's anything I ask that you don't want to answer, that is absolutely fine. Please let me know and I'll move on to the next question. Also, if you want to stop completely at any point that is okay. Just let me know. Does that all make sense?

I will start with a few questions about yourself.

A. ICE-BREAKERS

1. **Can you tell me how old you are?**
2. **What are your hobbies/interests?**
3. **Can you describe what a typical day is like for you?** *[prompts for morning/school/after school/evening].*

B. MY EMOTIONS AND ME: CREATIVE TASK

Description: In this task, the participant will present an art/craftwork that we have assigned for them to do at home before the session. We will discuss with the young people how this art/craftwork represents their emotions.

Instruction: *Now, we've asked you to make something to express the times that are calming, reassuring or difficult and upsetting or both, and reflect on your reactions. Shall we look at what you've made?*

1. ***What is it?***
   - *Please read/show/describe to me what you've written/made.*
   - *Does it have a name/title (e.g., X)?*
2. ***How does this (or X) relate to you being feeling this emotion?***
   - *What are the situations that X remind you of…?*
   - *What kind of emotion do you feel then?*
   - *What makes it emotional for you? How is it different from the usual emotion you have?*
3. ***How long do you feel that way?***
   - *Did it keep going? What kept it going?*
   - *Did the feeling change over time?*
4. ***How do these feelings stop?***
   - *Do they go away on their own?*
   - *Do you have to do anything to handle them? What are they?*
5. ***What else can you tell me about X?***

C. MY EMOTIONS AND ME: RESPONDING TO SCENARIOS

Description: Participants will be asked to select a vignette from a selection (of up to 8) presented on their screen. These will be displayed as coloured cards on the screen with a short title. Potential vignette topics are listed below. When the young person clicks on a card, a short video clip will play

Correspondence to: georgia.pavlopoulou@ucl.ac.uk ; edmund.sonuga-barke@kcl.ac.uk

(for 20-30 secs). In the clip, a young person with a diagnosis of ADHD and/or autism, will appear and briefly narrate a scenario. This will include captions. At the end of the video, a photo or drawing will appear inviting the participant to consider whether this situation is relevant to their own lives, and how this (or a similar situation) would affect them. We will stay curious about what it is like for the young person, and explore what the internal and external factors are in each situation.

We will then go through the questions below with the young person.

Example vignette

In school, we worked in pairs and each person had to write a summary of a text. On the day of the presentation, my partner hadn't done his part and blamed me, saying I didn't give him the correct information. (photo of a school classroom)

Instruction: *Ok, I will now ask you to look at different scenarios and think: whether you have ever been in similar situations; what your emotional reactions were; and how you managed (or didn't) to stop these emotions. Let's start with you choosing which scenario we would like us to start with by clicking on the box of your preference. Once you click on the box, a short clip will play presenting you with a scenario. I will ask you 5 questions for each card. Ready? (showing card/slide/vignette). There are no right or wrong answers.*

1. ***Is that situation familiar to you?***
   - Have you ever been in a situation where …
2. ***What's your emotional reaction in a similar situation?***
   - *How do you feel then? You can use the emotion chart to help describe your feeling.*
3. ***How do you express your emotion in that situation?***
4. ***What is your reaction to other people when you feel that way?***
5. ***What is the reaction of others to you when you feel that way?***
6. ***Have you ever managed not to feel that way?***
   - *Could you change your feelings once they have started? What had helped you to stop the feelings before they started?*
   - *Did your environment allow you or stop you to do what you needed to stop these feelings?*
7. ***How was it after you had experienced the feelings?***
   - *For example, some young people have told us they couldn't get to sleep that night thinking about this. What about you? What do you do after a similar situation? What would you do?*

C. MY EMOTIONS AND ME: THINKING ABOUT DIFFERENT EMOTIONS

Description: In this segment, we will show a colour-coded chart containing words (and/or emojis) that describe different clusters of emotions (see below). We will ask the young person to pick one emotion from each colour and go through the questions below. If appropriate, we could consider a participatory approach, e.g., by presenting the task in a playful manner, to help the young person access the interview. For instance, we can ask the young people to decorate the emoji or to match the emoji with a colour zone/emotion. We will make no prior assumption of what the chosen emotion means. Rather, we will stay open to the young person's definition. We will stay curious as to what it is like for the young person and explore what the internal and external factors are, i.e., check if the person is able to recognise traits in themselves and others/environment.

Correspondence to: georgia.pavlopoulou@ucl.ac.uk ; edmund.sonuga-barke@kcl.ac.uk

GREEN: Calm, Happy, Focused, Ready to Learn/ Play/Interact with others, Content, Excited

BLUE: Sad, Bored, Tired, Sick, Disappointed

YELLOW: Worried, Silly, Frustrated, Stressed, Hurt, In pain

RED: Mad, Upset/Loud, Scared, Angry, Out of Control, Irritable

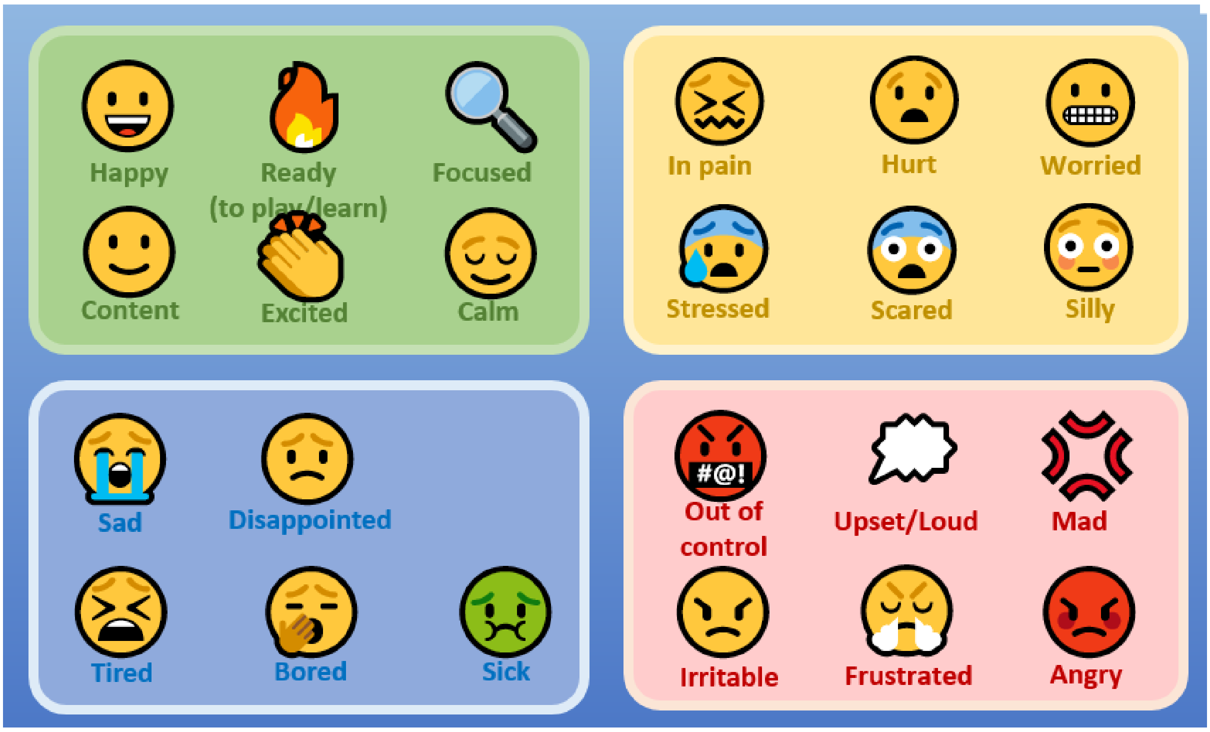

Figure S6. Emoji visual prompt used in interview

Instruction: *I will show you some words that express different emotions. I will ask you what situations bring these feelings at home, school or elsewhere, how long these feelings last, how you handle them and how you stop them. Let's pick a colour.*

1. ***So, what was it in your life at home or school that has made you feel X?***
   - *Or can you tell me about a time at home or school when you felt X?*
2. ***Are there times/occasions that you are more likely to feel X? Is it at home or school? Is it during or after an event?***

Useful additional general prompts (see the above for examples of when to ask the prompts):

- ➔ *How is this related to how you experience X?*
- ➔ *How does that make you feel?*
- ➔ *What do you do when you feel like this?*
- ➔ *Can you tell me about a time when…?*
- ➔ *Reflect by repeating a word and wait to hear clarification.*
- ➔ *Can you go back and tell me a little bit more about a time when…*
- ➔ *What do you mean by "bad"? Help me understand what "bad" looks like for you.*

Is there anything that I haven't asked you about X that you'd like to tell me?

Correspondence to: georgia.pavlopoulou@ucl.ac.uk ; edmund.sonuga-barke@kcl.ac.uk

(Discuss reimbursement with the YP - THANK YOU- REMIND SUPPORT CONTACT LINES)

Correspondence to: georgia.pavlopoulou@ucl.ac.uk ; edmund.sonuga-barke@kcl.ac.uk
